# EEG-based brain connectivity and sentiment analysis from smartphone social communication: insights into remitted major depressive disorder among adolescents

**DOI:** 10.1038/s44277-025-00044-x

**Published:** 2025-10-07

**Authors:** Nayoung Kim, Lilian Y. Li, Carter J. Funkhouser, Allison M. Letkiewicz, Esha Trivedi, Aishwarya Sritharan, Sarah Elizabeth Sarkas, Madeline Marie McGregor, Katherine Durham, David Pagliaccio, Alva Tang, Nicholas B. Allen, Stewart A. Shankman, Randy P. Auerbach

**Affiliations:** 1 Department of Psychiatry, Columbia University, New York, NY USA; 2 Department of Psychiatry, Northwestern University, Chicago, IL USA; 3 Department of Psychology, University of Texas, Dallas, TX USA; 4 Department of Psychology, University of Oregon, Eugene, OR USA

**Keywords:** Biomarkers, Depression

## Abstract

Although prior research has identified abnormal brain connectivity in remitted depressed adolescents, there is limited work associating these alterations with real-time affective dynamics, which may shed insight about specific biological markers that contribute to depression recurrence. Effective brain connectivity was estimated using renormalized partial directed coherence (rPDC) from resting-state EEG data collected in adolescents (N = 144; ages 13–18), including youth with remitted depression (n = 85) and healthy controls (n = 59). Additionally, over the course of 180 days, ~2.3 million messages from adolescents’ smartphones were passively obtained, and sentiment (i.e., words connoting positive and negative emotion) was extracted using the Python tweetNLP package. At the baseline and 6-month follow-up assessments, clinical interviews were administered to assess depressive symptom severity. Compared to healthy adolescents, youth with remitted depression exhibited hyperconnectivity in theta and delta frequency bands as well as hypoconnectivity in alpha, beta, and gamma in occipito-temporal regions (ps < 0.05). Across all participants, lower connectivity between the precuneus and middle temporal gyrus within the beta and gamma frequency bands was associated with greater negative sentiment in smartphone-based language (beta: B = −0.019, p = 0.006; gamma: B = −0.180, p = 0.007) but not depressive symptoms (beta: B = 0.073, p = 0.415; gamma: B = 0.093, p = 0.289). Conversely, lower alpha band connectivity from the mid cingulate cortex to the precuneus is associated with greater depressive symptoms at follow-up relative to baseline (B = −0.239, p = 0.013). These findings suggest that resting-state effective connectivity may serve as a neural marker of vulnerability for elevated depressive symptoms and negative affective expression during adolescence, highlighting potentially separable neurophysiological targets that, if replicated, could inform future preventive interventions.

## Lay summary

We studied brain activity in adolescents with and without a history of depression, and how it relates to their everyday emotional expression in text messages. Using EEG, we found that certain patterns of brain connectivity were linked to more negative language and to later increases in depressive symptoms. These findings suggest that specific brain activity patterns may serve as early warning signs for depression risk, helping guide future prevention efforts.

## Introduction

Major depressive disorder (MDD) is a major public health concern amongst adolescents, as approximately 20% will experience a depressive episode by age 18 [1]. Rates of depression are increasing in the United Sates, with the majority of adolescents experiencing recurrent episodes both during adolescence and across the lifespan, contributing to academic struggles, risky behavior engagement, psychiatric comorbidity, and heightened suicide risk [2, 3]. Adolescence is thus a critical period for both the onset and recurrence of depression, underscoring the urgent need to identify neural markers that signal vulnerability for depression. Although many adolescents achieve clinical remission, recurrence remains common, suggesting that symptom-based assessments alone may be insufficient to capture future risk.

Previous studies have suggested that affective vulnerability may manifest across both internal symptoms and external behavioral outputs, such as emotional expression in language [4–6]. Characterizing how neural connectivity supports both dimensions of affective functioning may enhance efforts to detect residual risk [6]. Resting-state brain connectivity has emerged as a promising candidate biomarker, as it can reveal persistent disruptions in brain networks even after symptoms have subsided. In particular, resting-state electroencephalography (EEG) provides a non-invasive and scalable method for measuring fast-changing neural dynamics implicated in emotion regulation and cognitive control—processes central to both the onset and recurrence of depression. EEG connectivity is commonly analyzed across canonical frequency bands—delta (1–4 Hz), theta (4–8 Hz), alpha (8–14 Hz), beta (14–30 Hz), and gamma (30–40 Hz)—each associated with distinct neurocognitive and affective functions. Delta and theta oscillations are linked to emotional regulation, self-referential thinking, and large-scale network integration, including activity within the default mode network (DMN) [7, 8]. Alpha is implicated in cortical inhibition and attentional control [9], while beta and gamma frequencies support semantic processing, language comprehension, and top-down regulation of emotion [10, 11].

Prior EEG studies in individuals with MDD and remitted MDD (rMDD) have identified frequency-specific alterations in connectivity that may reflect persistent disruptions in affective and cognitive processing systems. Elevated theta and alpha connectivity have been associated with increased self-referential processing and impaired emotion regulation [12, 13]. Disruptions in beta and gamma bands, particularly in frontotemporal and parietal regions, have been linked to deficits in language, memory, and social cognition [14–16]. Importantly, some of these neural abnormalities appear to persist after remission. Elevated theta-band coherence across the default mode, salience, and frontoparietal networks has been observed in adolescents with remitted MDD, suggesting lingering dysregulation in large-scale emotional control circuits [17]. Similarly, increased functional connectivity between the posterior cingulate cortex and subgenual prefrontal cortex has been identified in remitted individuals, pointing to a potential electrophysiological “scar” linked to rumination [18].

In the current study, we quantified brain connectivity using effective connectivity, which captures directional, time-sensitive interactions between brain regions—unlike functional connectivity, which reflects only undirected statistical associations. Specifically, we applied renormalized partial directed coherence (rPDC) [19], a multivariate frequency-domain method that estimates the flow of information between neural sources over time [20]. This approach is well-suited to EEG’s millisecond-level resolution and provides a mechanistic view of neural communication that may underlie both depressive symptoms and real-world emotional expression.

In parallel with clinician-rated symptom measures, we examined a real-world behavioral indicator of emotional expression: negative sentiment in adolescents’ daily smartphone text messages. As digital communication has become a primary mode of peer interaction during adolescence, recent studies have leveraged passively collected smartphone data to better understand behavioral patterns linked to MDD in youth [4]. With smartphone use being nearly universal among adolescents [10], these devices offer a unique, ecologically valid window into naturalistic language use—providing an opportunity to capture subtle linguistic markers of depression in everyday contexts. Importantly, emerging research has begun to link neural connectivity with real-world language behavior in adolescents [6]. For example, reduced connectivity between the salience network (SN) and central executive network (CEN) has been found to moderate the relationship between depressive symptoms and the use of negative emotion words during smartphone-based communication [6]. This finding suggests that everyday language may reflect underlying neurocognitive vulnerabilities associated with adolescent depression.

Building on this work, the present study integrates clinical, behavioral, and neural domains to examine whether resting-state effective connectivity patterns in adolescents with remitted MDD are associated with both (1) clinician-rated depressive symptoms and (2) emotional expression through digital communication. This dual-outcome framework supports our conceptualization of EEG-based connectivity as a latent risk marker that influences both internal mood states and their external expression. Therefore, the present study aimed to test whether adolescents with rMDD exhibit frequency-specific alterations in resting-state effective connectivity relative to healthy controls, and whether these patterns are associated with both future depressive symptoms and naturalistic negative sentiment expression in text messages. First, we hypothesized that rMDD youth would show increased delta, theta, and alpha connectivity in frontal regions, decreased alpha in parietal-occipital regions, and altered beta/gamma connectivity in temporoparietal areas—consistent with prior findings on emotion regulation, attention, and language processing [12–14, 17]. Second, we predicted that lower posterior alpha connectivity at baseline would be associated with greater depressive symptoms at 6-month follow-up. Last, we adopted an exploratory, data-driven approach to identify whether baseline effective connectivity patterns are also related to the use of negative sentiment in daily smartphone-based social messaging. By bridging neural and behavioral markers, we aim to clarify how brain network dynamics relate to risk for symptom recurrence and affective expression in naturalistic adolescent contexts.

## Methods and materials

### Participants

Participants were enrolled from September 2020 to June 2023, as part of an ongoing longitudinal project investigating social processing deficits in adolescent depression (for details, see [4, 16]). Briefly, adolescents ages 13–18-years-old were recruited from community and mental health clinics in the New York, NY and Chicago, IL areas. Inclusion criteria included: (a) Tanner Stage ≥3 [21], (b) proficiency in English, (c) Wechsler Abbreviated Scale of Intelligence-II (WASI-II) score ≥85 [22], (d) ownership of a personal smartphone (Android or iOS), and (e) right-handedness. General exclusion criteria included: (a) history of head injury, seizures, or other neurological disorders, (b) current moderate or severe substance use disorder, and (c) lifetime history of bipolar or psychotic disorders, oppositional defiant disorder, conduct disorder, organic mental disorder, or developmental disorder (e.g., autism).

Additional criteria were applied to the remitted depression group. These participants had a confirmed past episode of MDD, a Children’s Depression Rating Scale-Revised (CDRS-R) [23] score ≤54 at enrollment, and no current MDD episode or persistent depressive disorder. Further exclusions for this group included: (a) lifetime history of the psychiatric conditions listed above, (b) current high suicide risk, and (c) use of psychotropic medications other than antidepressants or stimulant medication. The final sample (N = 144) included remitted depressed adolescents (n = 85) and healthy controls with no lifetime history of psychiatric disorders (n = 59). See Table 1 for summary of sociodemographic and clinical characteristics.

**Table 1:** Clinical and Sociodemographic Characteristics Stratified by Group.

	Total Sample	Healthy Adolescents	Remitted Depressed Adolescents	t-/*χ*^2^ test	df	*p*
	N = 144	n = 59	n = 85			
**Age**		15.85 (1.47)	16.75 (1.48)	0.898	133.36	0.3708
**Sex**	105 (72.92%)	35 (59.32%)	70 (82.35%)	8.224	1	0.004
**Race**				5.618	5	0.345
White	70 (48.61%)	24 (40.68%)	46 (54.12%)			
Asian	25 (17.36%)	12 (20.34%)	13 (15.29%)			
Black/African American	18 (12.50%)	11 (18.64%)	7 (8.24%)			
American Indian/Alaska Native	1 (0.69%)	0	1 (1.18%)			
Native Hawaiian or other Pacific Islander	0	0	0			
More than one race	16 (11.11%)	6 (10.17%)	10 (11.76%)			
Unknown or Not reported	14 (9.72%)	6 (10.17%)	8 (9.41%)			
**Family income**				1.735	5	0.885
Less than $25,000	6 (4.17%)	1 (1.69%)	5 (5.88%)			
$25,000 – $50,000	18 (12.5%)	8 (13.56%)	10 (11.76%)			
$50,000 – $75,000	9 (6.25%)	4 (6.78%)	5 (5.88%)			
$75,000 – $100,000	23 (15.97%)	9 (15.25%)	14 (16.47%)			
$100,000 or more	63 (43.75%)	27 (45.76%)	36 (42.35%)			
Unknown or Not Reported	25 (17.36%)	10 (16.95%)	15 (17.65%)			
**Lifetime social anxiety disorder**	22 (15.28%)	–	22 (25.88%)			
**Lifetime generalized anxiety disorder**	26 (18.06%)	–	26 (30.59%)			
**Lifetime posttraumatic stress disorder**	11 (7.64%)	–	11 (12.94%)			
**Lifetime attention-deficit hyperactivity disorder**	14 (9.72%)	–	14 (16.47%)			
**Lifetime substance use disorder**	3 (2.08%)	–	3 (3.53%)			
**Smartphone type**				0.923	1	0.337
iOS	126 (87.5%)	54 (91.53%)	72 (84.71%)			
Android	18 (12.5%)	5 (8.47%)	13 (15.29%)			
**Site**				18.36	1	<0.001
New York	83 (57.64%)	47 (79.66%)	36 (42.35%)			
Chicago	61 (42.36%)	12 (20.34%)	49 (57.65%)			

### Procedure and materials

All study procedures adhered to the Declaration of Helsinki and were approved by the New York State Psychiatric Institute Institutional Review Board. Informed assent and consent were obtained from minor adolescents and their parents, respectively, and 18-year-old adolescents provided informed consent. Participants were administered the WASI-II (two-subtest form) to evaluate verbal intelligence (Vocabulary subtest) and nonverbal intelligence (Matrix Reasoning subtest). Then, participants installed the Effortless Assessment Research System (EARS) app [24] on their personal smartphones to obtain keyboard inputs over a 6-month period. In this study, keyboard inputs refer to text (e.g., words, emojis) from a participants’ typical smartphone use, including real-world typing in messaging and other digital interactions (e.g., text messages, social media posts). Participants were instructed to keep the app active while using their phones as usual. The EARS app was configured to run in the background, automatically collecting data without requiring manual intervention. If the app was closed or its background activity was interrupted, this was discernible from the data logs, which recorded gaps in data collection. There were two primary technical reasons for missing text data. First, some participants chose not to install the EARS keyboard or later uninstalled it due to usability issues, resulting in partial or full absence of language data. A small subset also opted out of language tracking entirely. Second, both iOS and Android operating systems automatically disable unused apps (after ~12 days for iOS and ~3 months for Android). When the EARS app was deactivated in this manner, all data collection—including keyboard input—ceased until the app was reactivated, typically following study team outreach. These sources of missingness were unrelated to study conditions and stemmed from technical and behavioral compliance factors. On average, participants contributed usable text data for 162.07 days (SD = 17.02) out of the 180-day (6-month) observation period. There was no significant difference in the number of days with valid sentiment data between adolescents with remitted MDD and healthy controls (t(142) = 0.368, p = 0.713), suggesting comparable data availability across groups. These findings indicate that missingness did not systematically differ by diagnostic status and that language data coverage was robust across the sample.

In a separate session, resting state EEG data were acquired. Clinical interviews assessing depressive symptoms were re-administered at the 6-month follow-up assessment.

### Clinical interviews

During the initial lab visit, participants were administered the Kiddie Schedule for Affective Disorders and Schizophrenia (K-SADS-PL; [25]) to evaluate lifetime psychiatric disorders (k = 0.98). At the baseline and the 6-month follow-up assessment, participants also completed the CDRS-R, a 17-item interview assessing depressive symptom severity. The total score on the CDRS-R ranges from 17 to 113, with higher scores indicating greater severity of depressive symptoms.

### Electrophysiological recordings and data reduction

Resting-state EEG (3-minutes eyes closed) data were acquired using the 32-channel ActiCHamp from Brain Products (Brain Products, Munich, Germany) positioned according to the 10–20 international system using the BrainVision Recorder. The data were digitized at a 500 Hz sampling rate and referenced online to FCz. The ground electrode was placed between electrodes Fp1 and Fp2. Vertical and horizontal EOG data were recorded, and electrode impedances were maintained below 20 K ohms. For details regarding the preprocessing procedures, see the Supplement.

### Effective connectivity analysis

EEG preprocessing and source analysis were performed using the EEGLAB and groupSIFT toolboxes [26]. After artifact rejection and re-referencing, we applied infomax independent component analysis (ICA) to decompose EEG signals into temporally independent sources. Equivalent dipole models were estimated for each component using a standard boundary element head model. Components with dipole locations outside the brain or residual variance >15% were excluded [20, 27, 28]. Only dipoles localized within cortical gray matter and outside artifact-prone regions (e.g., eyes, neck) were retained. To enable group-level analysis, we used the groupSIFT framework to cluster dipoles into anatomically defined brain regions based on the AAL atlas [27, 28]. For each participant, ICA identified multiple independent components (bounded by data rank), each localized with DIPFIT (dipole-fitting toolbox). Brain-source ICs were retained. Across participants, this yielded a large pool of brain-source ICs, from which we computed the grand-mean IC×IC effective connectivity separately for each group. This method allows for the aggregation of component time series into common spatial nodes, making it possible to compare effective connectivity patterns across participants. This approach allows for source-level connectivity estimation even in low-to-moderate density EEG recordings [27–29]. Although high-density EEG offers finer spatial resolution, several studies have shown that meaningful connectivity dynamics can be extracted using GroupSIFT from datasets with as few as 20–40 channels [27–29]. These findings support the robustness of our pipeline for identifying meaningful connectivity dynamics even with limited spatial resolution.

#### IC-space effective connectivity

Using groupSIFT, we estimated rPDC in the IC×IC space to quantify directed, frequency-resolved connectivity among retained ICs. The rPDC served as a weighting factor during this transformation, accounting for dipole density contributions across participants. The rPDC was selected for its capacity to capture directional connectivity in multivariate time series, which is advantageous for exploratory EEG studies. Unlike dynamic causal modeling (DCM), which provides detailed biologically informed modeling, rPDC operates without the need for participant-specific anatomical data such as MRIs, offering a more accessible and versatile approach in the context of EEG. Furthermore, rPDC is uniquely suited to high-temporal-resolution EEG data due to its computational efficiency and adaptability in handling complex, non-stationary signals, enabling a comprehensive analysis of dynamic brain connectivity patterns.

#### GroupSIFT source-space projection

Inference was performed in source space rather than at the sensors. To place ICs into a common anatomical frame, each IC’s dipole coordinate was convolved with a 3-D Gaussian kernel (FWHM = 20 mm) to generate a probabilistic dipole-density field [20, 29]. Dipole density within brain space was then segmented into a modified AAL atlas (76 nodes) in which subcortical labels are consolidated into upper basal and lower basal groups to avoid over-interpreting specific subcortical nuclei from scalp EEG (mitigating depth bias in single-dipole fits and the spatial extent of cortical generators) [29, 30]. Using the resulting region-wise density weights, IC → IC rPDC was projected to regions of interest (ROI) → ROI by weighting each connection according to the probability that its source IC belongs to ROI i and its target IC to ROI j. This procedure yields subject-consistent, atlas-indexed connectivity matrices and resolves post-ICA inter-subject variability in IC numbering and location.

#### Group-level node definition

Because analyses occur in source space, multiple ICs from a given participant can contribute to multiple regions. To ensure stability at the group level, we retained only regions with broad coverage—nodes to which ≥65% of participants contributed at least one cortical dipole. This threshold was chosen to balance the need for adequate representation of participants and the reliability of data across nodes, guided by thresholds commonly employed in similar studies [26, 27] and informed by considerations of data quality. Of the 76 atlas nodes, 47 met this criterion and were used for all ROI-level analyses. These 47 nodes captured 80.2% of the total cortical dipole-density mass across participants, indicating that selected nodes were reliably represented in the sample. This serves as a proxy measure to ensure the nodes selected for connectivity analysis were reliably represented across participants. Subsequently, connectivity metrics were derived exclusively from these 47 shared nodes, facilitating robust and interpretable group comparisons.

#### Statistical analysis

To identify group differences in effective connectivity, we computed pairwise rPDC between all retained cortical nodes (N = 47) across five frequency bands. This resulted in 1081 directed connections per band. Given the large number of comparisons, we used a cluster-level permutation test implemented in the groupSIFT toolbox to control the family-wise error rate (FWER) at p < 0.05 [27, 28]. Specifically, we applied a nonparametric approach using 2000 label shuffles with cluster correction based on spatial adjacency across nodes. This provides a weak FWER correction, balancing sensitivity and specificity when large-scale network patterns are of primary interest. For further details on this workflow and tool application, see prior work [26, 27] and consult the GroupSIFT repository for practical guidelines and software setup.

### Key input data and sentiment analysis

To capture naturalistic emotional expression, we collected keyboard input data using the EARS app (Effortless Assessment Research System; Ksana Health Inc.), which was installed on participants’ personal smartphones. The app includes a custom keyboard that passively records keypresses across all applications, capturing only self-generated text. Incoming messages and third-party content were not collected. All keystrokes, including deletions (e.g., backspaces), were time-stamped, locally encrypted, and transmitted to a HIPAA-compliant cloud server. Additional privacy and security protocols are detailed in the Supplement. Each keystroke was labeled with metadata indicating the active app when available. Due to iOS 16+ restrictions, app metadata was missing for approximately 11.4% of messages. Among the remaining messages, 81.8% were generated in social communication apps, with Apple Messages, Instagram, and Snapchat being the most frequently used.

#### Message reconstruction and preprocessing

Raw keystroke data were reconstructed into complete messages using a custom Python pipeline [5]. Logical message boundaries were defined by pauses ≥5 s, app switches, or return/enter key usage. In addition, the EARS platform segmented messages based on either (1) the first entry in a given app or (2) a string with <50% overlap with the preceding message. We removed partial or redundant strings—defined as messages entirely contained within the subsequent message and typed in the same app within 60 s—to reduce noise. Of the 144 participants included in the analysis, 126 (87.5%) used iOS devices and 18 (12.5%) used Android. Typed text was recorded uniformly across platforms. On Android devices, system-generated messages (e.g., ‘Send message…’) were occasionally recorded. These were identified based on duplication, structure, or trailing ellipses and manually removed during preprocessing. No such artifacts were observed in iOS data. A message was retained if it contained more than one character, unless it was a single-character emoji. No minimum word count was required, as the TweetNLP model is optimized for short-form digital content including single words and emojis.

#### Text cleaning and sentiment classification

Text data were preprocessed to optimize sentiment classification while preserving emotional nuance. Following best practices, we: (a) Removed URLs and user mentions (e.g., “@username”); (b) Normalized elongated words (e.g., “noooo” → “no”); (c) Retained punctuation, capitalization, and emojis to preserve affective cues; and (d) Translated non-English messages (0.3%) using Google Cloud’s Translation API, as TweetNLP supports English only. Initial preprocessing scripts included hashtag segmentation (e.g., “#joytotheworld” → “joy to the world”), a step adopted from pipelines using lexicon-based tools like VADER. However, TweetNLP—trained on 124 million tweets—is robust to social media syntax, including hashtags and emojis. We verified that sentiment labels remained consistent with and without hashtag segmentation, so hashtags were retained in their original form for final analyses.

#### Sentiment metrics and data coverage

Sentiment classification was conducted using the TweetNLP Python package [31], which includes a RoBERTa-based transformer model fine-tuned on tweets from 2018–2021. Each message was assigned a single sentiment label (positive, negative, or neutral) using the tweetNLP classifier, which detects the dominant sentiment based on contextual cues. Although some messages may contain mixed emotional tones, the model assigns the sentiment that is most strongly conveyed. Future work could benefit from multi-label or emotion-specific models to better characterize nuanced affective content.

For each participant, we computed the daily proportion of positive and negative messages (relative to total daily messages), and averaged these proportions across the 6-month follow-up period to generate person-level sentiment metrics. To account for individual differences in overall expressivity and emotional valence, daily proportions of positive sentiment were included as covariates in analyses examining negative sentiment.

### Data analytic approach

For smartphone communication data, we included participants who had sent at least 3 messages per day and had data available for at least 7 days within the initial 180-day period. Among the 144 participants, 18 individuals (7 remitted depressed adolescents, 11 healthy controls) were excluded due to a lack of EEG collection. Additionally, 5 participants (2 remitted depressed, 3 healthy controls) were further excluded due to poor EEG data quality. Thus, the final analysis included a total of 121 participants (76 remitted depressed, 45 healthy controls). We used a two-stage approach: (i) baseline group differences (discovery): cluster-based permutation testing with omnibus FWER control across time × frequency × edges; and (ii) predicting depressive symptoms and sentiment in language (targeted associations): theory-constrained outcome models with within-outcome FDR.

#### Baseline group differences

Cluster-level correction was employed to control the generalized familywise error rate using permutation tests (i.e., to identify which connectivity effects remained statistically significant) [26]. To generate surrogate statistics representing the null hypothesis (i.e., no group effect), the group labels of renormalized partial directed coherence arrays were iteratively shuffled (N = 1000). Statistical comparisons were then repeated for each iteration and each graph edge.

#### Predicting sentiment in language and depressive symptoms

**Fig. 1:**
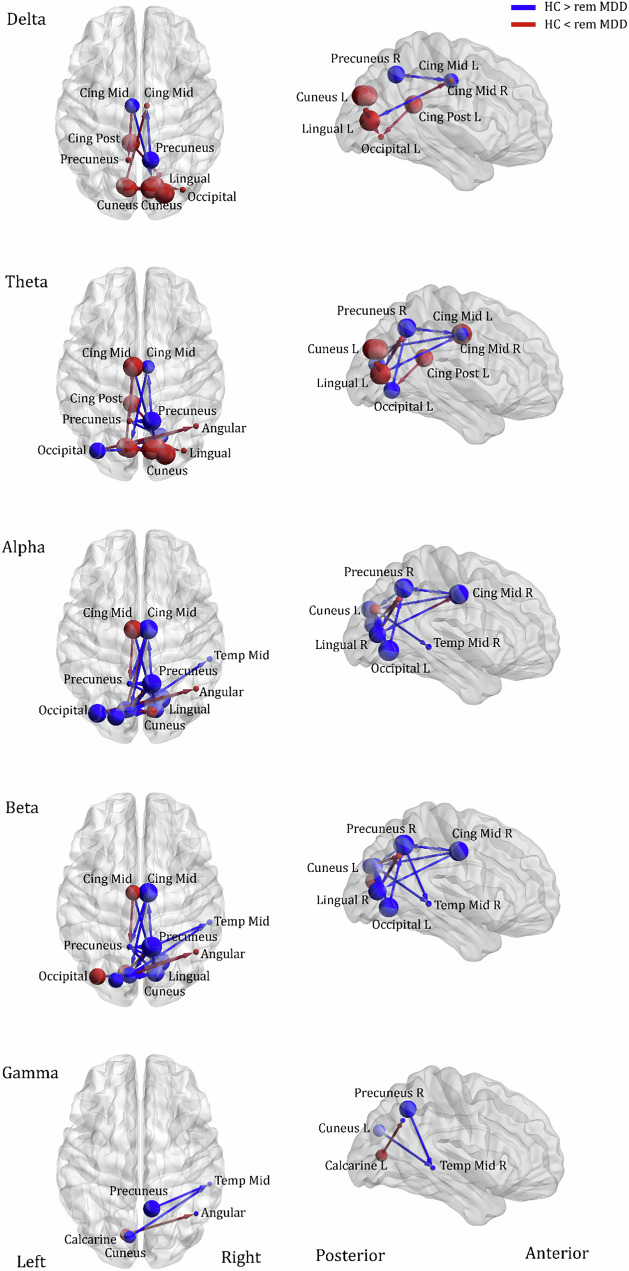
Effective connectivity of resting-state EEG activity in healthy and remitted depressed adolescents. Red arrow indicates high effective connectivity and blue arrow indicates low connectivity in remitted depressed adolescents relative to healthy comparison subjects. Sphere size indicates the amount of total outflow in each node. remMDD, adolescents with remitted major depressive disorder; HC healthy controls.

To select specific connections for the association analysis between EEG effective connectivity and depressive symptoms as well as sentiment in language, we focused on region-to-region connections, guided by our hypothesis and previous findings. For depressive symptoms at the 6-month follow-up, we focused on one a priori hypothesized pathway: effective connectivity between the mid-cingulate cortex (MCC) and the precuneus in the alpha band, based on prior studies linking this connection to internally directed thought and depressive rumination [12, 13, 17]. To assess frequency specificity, we also tested the same MCC–precuneus edge in delta, theta, and beta bands. Similarly, for negative sentiment in daily smartphone messaging, we selected region-to-region connections within each band that both (1) showed significant group differences, and (2) involved regions relevant to semantic and affective processing (e.g., middle temporal gyrus, precuneus) [6, 7, 32]. These edges were chosen to ensure theoretical interpretability and limit model complexity. Across all edges, the second-largest F-statistic-weighted cluster was extracted in each iteration to generate a null distribution for each effect. This approach, consistent with prior research emphasizing network-level effects, helps mitigate the risk of spurious findings driven by single large clusters. The true F-statistic-weighted mass of clusters was then compared to this null distribution, with significance determined at p < 0.05. Only significant results, which are depicted in Fig. 1, were included in subsequent analyses.

We tested whether baseline resting-state connectivity was associated with the negative sentiment of smartphone social communication, adjusting for the covariates of positive sentiment, age, sex, site, and phone type. Additionally, a negative binomial regression model using generalized estimating equation analysis of repeated measures estimated the association between resting state connectivity and depression symptoms at the 6-month follow-up assessment, while adjusting for baseline depression severity, age, sex, and site.

Although negative sentiment and depressive symptoms are related constructs, we did not include baseline depression as a covariate in the sentiment model in order to preserve meaningful behavioral variance in linguistic expression. Negative sentiment in typed messages may reflect early emotional dysregulation or day-to-day affective tone that is not fully captured by retrospective symptom ratings.

## Results

### Baseline group differences

The connectivity matrix representing the group-difference (remitted depressed versus healthy subjects) of each EEG band activity (a predefined p < 0.0001, corrected; two-tailed [27]) is shown in Figure S1. The results revealed a number of significant hubs for effective connectivity (i.e., increased or decreased EEG phase coherence between two cortical regions) for each band: 11 for delta (1–4 Hz), 20 for theta (4–8 Hz), 25 for alpha (8–14 Hz), 23 for beta (14–30 Hz), and 3 for gamma activity (30–40 Hz).

#### Delta band activity (1–4 Hz)

Compared to healthy youth, remitted depressed adolescents exhibited decreased effective connectivity from a region near the right precuneus to the right MCC, and from the left MCC to the right calcarine gyrus (Fig. 1). Additionally, there was greater connectivity between the right and left cuneus and occipital areas observed in the remitted depressed group relative to the control group.

#### Theta band activity (4–8 Hz)

The overall pattern of theta connectivity was similar to delta band activity. Remitted depressed adolescents displayed a similar pattern of greater right hemisphere connectivity flow in the occipital area compared to healthy controls (Fig. 1). Relative to controls, remitted youth also showed decreased effective connectivity from a region near the right precuneus to the right MCC, as well as increased interaction between the left MCC and left precuneus. Notably, greater effective connectivity was observed from the left posterior cingulate cortex to the right lingual gyrus in the remitted depressed adolescents compared to the controls.

#### Alpha band activity (8–14 Hz)

Connectivity alterations were observed among the temporal, middle cingulate, and occipital regions in remitted depressed adolescents compared to healthy controls. These networks overlapped across beta and alpha band activities. Specifically, greater effective connectivity was observed from the left cuneus to the right middle temporal gyrus (MTG) in controls than in remitted depressed adolescents. Unlike at lower frequencies (<8 Hz), there was greater connectivity from occipital nodes (lingual gyrus) to the precuneus and MCC in controls compared to remitted depressed adolescents.

#### Beta band activity (14–30 Hz)

Similar to the alpha band, remitted depressed adolescents showed weaker effective connectivity from the right MCC to the left cuneus compared to healthy controls. Additionally, there was significantly greater connectivity from both the precuneus and cuneus to the MTG was found in controls compared to the remitted depressed group.

#### Gamma band activity (30–40 Hz)

Controls exhibited greater connectivity from both the precuneus and cuneus to the MTG compared to the remitted depressed group. On the other hand, greater connectivity from the left calcarine to the right angular gyrus was observed in the remitted depressed group compared to controls.

### Predicting depressive symptoms and smartphone communication sentiment

Interestingly, reduced alpha-band effective connectivity from the MCC to precuneus was associated with greater depressive symptoms at the 6-month follow-up (b = −0.239, SE = 0.096, p = 0.013) while controlling for baseline depression severity, age, sex, and site (Table 2). The standardized effect size for the association between alpha-band connectivity and depressive symptoms was Cohen’s d = −0.143, indicating a small to moderate association. Although the model’s R² was 0.323, reflecting the combined explanatory power of all predictors, including baseline symptoms and covariates, alpha-band connectivity remained a statistically significant predictor even when accounting for baseline symptoms, age, sex, and site; this suggests its potential utility as a neural marker. However, no significant associations were found between alpha-band connectivity (MCC to precuneus) and negative sentiment (b = 0.003, SE = 0.007, p = 0.712). Supplementary analyses showed that this relationship was not observed in delta, theta, or beta bands (see Supplementary Table 1), supporting the frequency specificity of the effect. We did not examine the gamma band for this connection due to the absence of reliable MCC dipole representations in that frequency range.

**Table 2:** Alpha band connectivity strength from mid cingulate cortex to precuneus predicting depression symptoms at the 6-month follow-up assessment.

	Estimate	SE	z value	*p*-value
Intercept	1.976	0.501	3.944	<0.001
Age	−0.004	0.024	−0.159	0.873
Sex	−0.166	0.084	−1.969	0.050
Site	−0.049	0.071	−0.684	0.494
Baseline depressive symptoms	0.024	0.007	3.371	0.001
Baseline connectivity (MCC→Precuneus)	−0.239	0.096	−2.490	0.013

Children’s Depression Rating Scale = Depressive symptoms; *MCC* mid cingulate cortex.

We also conducted a correlation analysis to examine the relationship between negative sentiment and CDRS scores. The results revealed a significant positive correlation (r = 0.310, p = 0.001), suggesting that higher levels of negative sentiment are associated with more severe depressive symptoms. Research has consistently shown that individuals with depression tend to exhibit lower positive sentiment and higher negative sentiment in their social media posts (e.g., Facebook, Twitter), effectively distinguishing those with depression from those without [33–35].

**Fig. 2:**
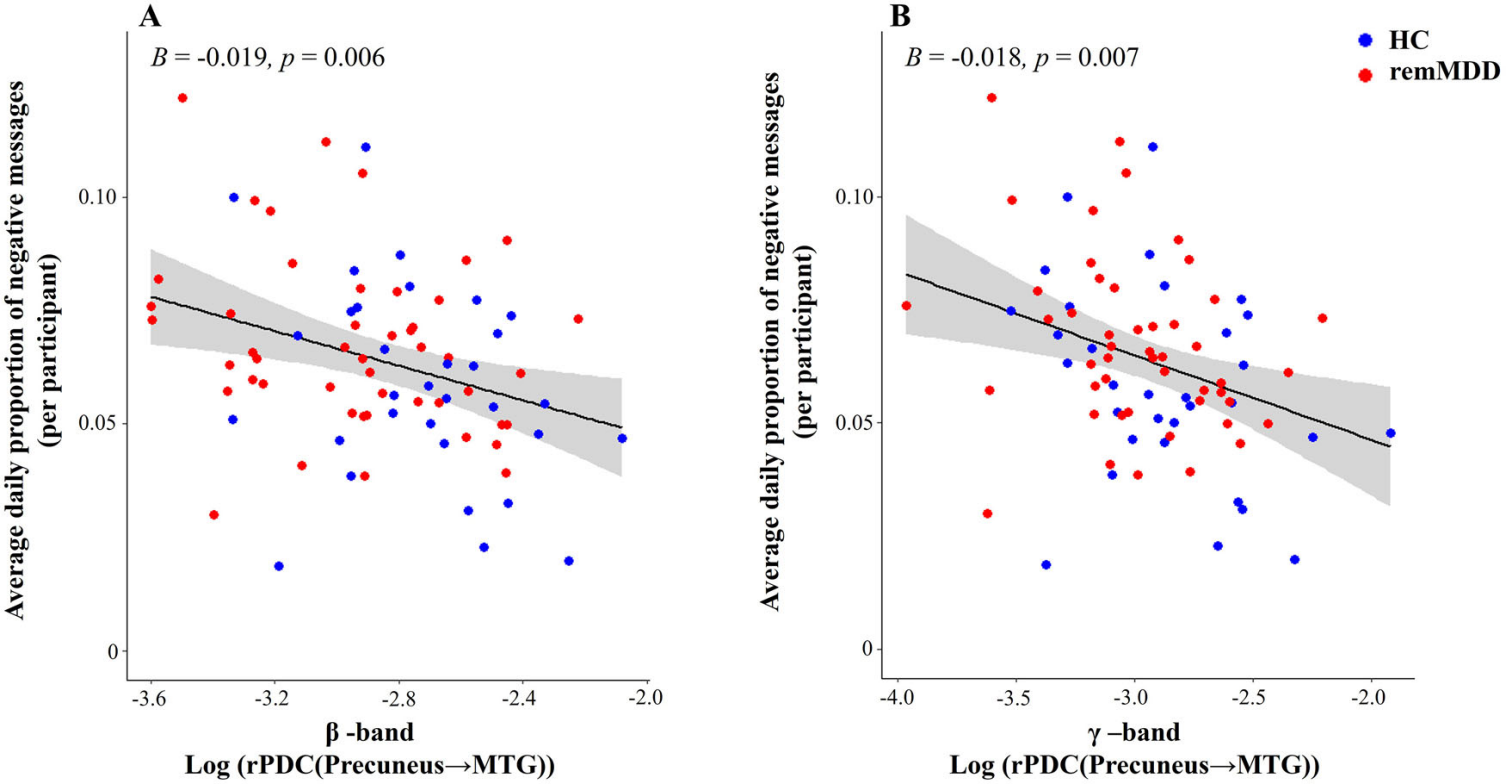
Association between resting-state effective connectivity and negative sentiment expressed in adolescents’ smartphone communication. Effective connectivity is represented by the log-transformed renormalized Partial Directed Coherence (log(rPDC)) from the precuneus to the middle temporal gyrus (MTG). Negative sentiment was computed as the daily proportion of negative messages (relative to total daily messages), averaged across the 6-month follow-up period to yield person-level metrics. **A** Beta-band connectivity. **B** Gamma-band connectivity. HC healthy controls, remMDD adolescents with remitted major depressive disorder.

Based on the strength of connectivity observed in the baseline analysis, we also examined its association with negative sentiment in adolescents’ smartphone social communication over a 6-month period. As shown in Fig. 2, reduced beta-band connectivity from the precuneus to the MTG was associated with more negative sentiment (Fig. 2A: b = −0.019, SE = 0.007, p = 0.006), covarying for positive sentiment, age, site, sex, and phone type. Gamma-band connectivity exhibited a similar pattern, as lower connectivity between the precuneus to the MTG was associated with more negative sentiment (Fig. 2B: b = −0.018, SE = 0.007, p = 0.007), adjusting for covariates (Table 3). There were, however, non-significant relationships in other bands, including: alpha (cuneus to MTG), theta (precuneus to MCC), and delta (precuneus to MCC; Table 3). Sensitivity analyses indicated that there were no significant associations between connectivity from precuneus to MTG in the beta-band (b = 0.073, SE = 0.089, p = 0.415) or in the gamma band (b = 0.093, SE = 0.088, p = 0.289) and depression severity at the 6-month follow-up assessment.

**Table 3:** Baseline connectivity magnitude predicts negative sentiment in smartphone social communication.

	Delta-band (Precuneus→MCC)			Theta-band (Precuneus→MCC)			Alpha-band (Cuneus→MTG)			Beta-band (Precuneus→MTG)			Gamma-band (Precuneus→MTG)		
	b	SE	*p*	b	SE	*p*	b	SE	*p*	b	SE	*p*	b	SE	*p*
(intercept)	0.045	0.027	0.095	0.045	0.027	0.097	0.020	0.033	0.511	0.006	0.030	0.838	0.007	0.030	0.817
Baseline connectivity	−0.286	0.563	0.613	−0.184	1.048	0.860	−0.005	0.006	0.389	−**0.019**	0.007	0.006	−**0.018**	0.007	0.007
Age	0.001	0.002	0.590	0.001	0.002	0.614	0.001	0.002	0.539	−0.0003	0.002	0.816	−0.0004	0.002	0.794
Sex	−0.007	0.005	0.219	−0.007	0.005	0.162	−0.001	0.006	0.853	−0.003	0.006	0.590	−0.001	0.006	0.857
Site	−0.001	0.005	0.770	−0.001	0.005	0.822	−0.001	0.005	0.776	−0.0005	0.005	0.915	<0.001	0.005	0.983
Phone type	0.009	0.006	0.159	0.009	0.006	0.163	0.007	0.007	0.272	0.008	0.006	0.203	0.008	0.006	0.224
Positive sentiment	0.008	0.040	0.840	0.008	0.040	0.848	0.027	0.043	0.531	0.021	0.041	0.608	0.024	0.041	0.555

*MCC* mid cingulate cortex; *MTG* middle temporal gyrus.

Bold values indicate statistical significance *p* < 0.05.

## Discussion

The present study investigated altered brain connectivity patterns across various frequency bands in adolescents, comparing those with remitted depression to healthy controls. Using a multivector autoregressive modeling approach, we found that remitted depressed youth exhibited hyperconnectivity in lower frequency bands (delta and theta) and hypoconnectivity in higher frequency bands (alpha, beta, gamma), particularly in occipito-temporal regions. Additionally, we examined associations between connectivity differences and two key outcomes at the 6-month follow-up period: (a) future depressive symptoms and (b) negative sentiment in smartphone social communication. These findings are consistent with prior research suggesting that depression is linked to disruptions in resting-state EEG connectivity. Specifically, individuals with MDD often show increased theta coherence and reduced alpha and beta connectivity, indicating impaired top-down regulation and cognitive control [36–38]. The hyperconnectivity in delta and theta bands observed in the remitted group may reflect residual deficits from prior depressive episodes [37, 38], while reduced connectivity in faster frequency bands may may compromise the modulation of cognitive and inhibitory processes [37, 39], contributing to difficulties in filtering out irrelevant information and focusing attention [9, 40, 41].

This study also tested the association between resting-state brain connectivity and future depressive symptom severity in adolescents. We found that weaker alpha-band connectivity between the MCC and the precuneus, key nodes within the DMN, was associated with an increase in depressive symptoms at a 6-month follow-up. Alpha-band oscillations, often associated with attentional processes, have been linked to the DMN role in self-referential thought and emotional processing [7]. Our findings suggest that altered communication within this network, as reflected by decreased alpha-band connectivity, may contribute to depressive symptoms at follow-up [32]. At follow-up, 9.4% of participants (n = 8) met or exceeded the clinical cutoff score (CDRS-R ≥ 55), suggesting that a subset may have experienced clinically significant symptom recurrence—underscoring the importance of identifying neural markers of risk even within a remitted sample.

Additionally, this study explored the association between brain connectivity and smartphone social communication patterns across all participants. Among all frequency bands and connectivity edges, we found that lower connectivity in the beta and gamma bands between the precuneus and the MTG at baseline was associated with greater negative sentiment in smartphone communication over the following six months. Previous research has also implicated reduced functional connectivity within the temporal and occipital lobes in the pathophysiology of depression [42]. Beta and gamma waves are associated with active information processing, focus, and higher cognitive functions such as language comprehension [43, 44]. The MTG plays a critical role in deciphering the meaning of words, social cues, and regulating emotions [45, 46], while the precuneus is crucial for mental imagery and empathy [47, 48]. A previous fMRI study found activation in both the precuneus and the MTG when respondents had to make empathic judgments in a verbal task [49, 50]. Accordingly, weaker connectivity between these areas may reflect alterations in the communication pathways involving regions critical for understanding others’ emotions and interpreting social cues. Furthermore, a recent fMRI study identified that lower connectivity between the SN and the CEN moderated the association between depression and negative emotion word usage in adolescent smartphone communication [6].

Together, these findings highlight distinct yet overlapping roles of DMN connectivity in internalizing symptoms and external emotional expression. Specifically, while MCC–precuneus connectivity was associated with depressive symptom severity, precuneus–MTG connectivity was linked to the emotional tone of adolescents’ digital communication. This pattern suggests a broader vulnerability at the network level that spans both affective experience and its expression [51]. Notably, both outcomes were associated with resting-state effective connectivity within overlapping neural systems—particularly the precuneus—supporting a unifying hypothesis: that disruptions in DMN and semantic-affective pathways may underlie both internalizing symptoms and real-world emotional communication in adolescents at risk for depression recurrence [51, 52].

Although these findings provide preliminary evidence for the potential of EEG-based connectivity analysis in understanding emotional processing and regulation in remitted depressed adolescents, there are notable limitations. First, one important limitation of the present study is the use of a 32-channel EEG system, which inherently restricts the spatial resolution of source localization. Although this configuration does not support precise mapping of cortical generators, we addressed this constraint by applying ICA-based source separation and estimating equivalent dipoles. These dipoles were then grouped into standardized brain regions using the groupSIFT pipeline, a method that has been validated for use with low-density EEG systems [27–29]. This approach enabled us to examine large-scale connectivity patterns across participants while minimizing the risks of spurious localization. Nonetheless, future studies employing high-density EEG or multimodal imaging approaches (e.g., EEG-fMRI) are warranted to improve spatial precision and further validate these findings. Second, our study relied on resting-state data rather than task-based data, which limits our understanding of the specific psychological process implicated by the EEG signal. Task-based data could potentially offer more robust insights into the functional dynamics of brain regions involved in social communication and emotional regulation. Third, the assessment of depressive symptoms is not as temporally fine-grained as the collection of smartphone communication data. Symptoms were evaluated at the 6-month assessment, whereas smartphone communication was continuously monitored during this period. Aligning these assessments more closely in future studies could provide a clearer picture of the relationship between real-time behavioral data and depressive symptomatology. Fourth, although our findings highlight the relevance of MTG and MCC in predicting depressive symptoms and negative sentiment across the entire sample, it is important to acknowledge the potential value of exploring group-specific patterns. The unique composition of our sample, which includes both remitted MDD youth and healthy controls, offers an opportunity to examine whether individuals with a history of depression exhibit distinct connectivity–symptom associations that may reflect residual vulnerability. Although the current analysis did not reveal significant group-by-connectivity interactions, this may be due to limited statistical power given the modest subgroup sizes. Future studies with larger samples are needed to clarify whether these neural patterns differentially characterize youth with prior depression, ultimately informing more personalized approaches to intervention. Additionally, the current study is the gender imbalance between groups, which reflects the higher prevalence of adolescent depression in females. Although all analyses controlled for gender, caution is needed when generalizing these findings. Last, test-retest reliability was not explicitly assessed in this study, which we acknowledge as a limitation. Although prior research has demonstrated moderate-to-high test-retest reliability for resting-state EEG connectivity metrics, particularly within similar frequency bands and analytic frameworks [53, 54], the stability of these connectivity patterns over time remains an important consideration.

Despite these limitations, this study provides novel evidence linking resting-state EEG connectivity to both prospective depressive symptoms and naturalistic emotional behavior, suggesting its potential utility as a transdiagnostic risk marker. Specifically, connectivity patterns among the MCC, precuneus, and MTG emerged as candidate predictors of both internal symptoms and externally expressed affect. These results underscore the possibility that how adolescents feel and how they communicate emotion in everyday life may be supported by shared neural mechanisms. Importantly, the inclusion of passively collected smartphone language provides an ecologically valid, scalable behavioral marker that complements traditional clinical assessments. Within this framework, our findings point to future opportunities for integrated brain–behavior monitoring using mobile and wearable technologies.

In sum, this study highlights a convergent set of neurophysiological features—including MCC–precuneus and precuneus–MTG connectivity—that may underlie adolescent vulnerability to persistent emotional difficulties. These connectivity patterns were associated not only with clinician-rated symptoms but also with adolescents’ real-world emotional language. This dual-outcome approach illustrates the promise of combining EEG-based biomarkers with digital behavioral signals to support early detection and targeted intervention. By focusing on neural systems that contribute to both emotional experience and expression, future tools may better identify and assist youth at heightened risk for ongoing affective challenges. Therapeutic interventions aimed at enhancing connectivity within specific brain networks, such as neurofeedback or transcranial magnetic stimulation, could be explored to address the underlying neurobiological mechanisms contributing to depression recurrence.

## Supplementary information

Supplemental Material

## Data Availability

Data are publicly available through the National Data Archive.
