# Supplementary material for: EEG-based brain connectivity and sentiment analysis from smartphone social communication: insights into remitted major depressive disorder among adolescents
Source: NPP Digit Psychiatry Neurosci. 2025 Oct 7;3:23. doi: 10.1038/s44277-025-00044-x (PMC12504114; doi:10.1038/s44277-025-00044-x)
Supplement: Supplementary file 1 — Supplemental Material [file 44277_2025_44_MOESM1_ESM.docx]

**Supplemental Material**

**Ethical Considerations and Data Security**

The New York State Psychiatric Institute Institutional Review Board approved all study procedures. Written informed assent and consent were obtained from participants aged 13–17 and their legal guardians, while participants aged 18 provided written consent. Significant ethical considerations arise when collecting mobile sensing data. In this study, passive smartphone data were gathered using the Effortless Assessment Research System (EARS) app [[1,2]](https://www.zotero.org/google-docs/?VNoRDZ), developed and maintained by Ksana Health.

Participants received both verbal and written information about EARS before providing consent or assent. The data collected by EARS were encrypted and stored on a cloud-based server. The authors then downloaded and decrypted the data. Only the study team had access to the data, and other parties involved in data collection and storage, such as Ksana Health, did not have access. Data collection involved a logging tool that recorded participants' self-generated keyboard inputs. No third-party keyboard inputs were recorded. Detailed information on the measures taken to protect participants’ privacy can be found in [[1,2]](https://www.zotero.org/google-docs/?TKpgOW), and on the Ksana Health privacy policy page. Deidentified data from this study were submitted to the National Data Archive (NDA) in accordance with the National Institute of Mental Health's Data Sharing Policy, which permits the sharing of deidentified research data while ensuring strict confidentiality for participants. To further safeguard privacy, all shared data were carefully reviewed to exclude any content that could potentially compromise third-party privacy. To protect participants' confidentiality, 'raw' key input data, which can include potentially identifying information (e.g., names, social media handles, and uncommon word strings), were not submitted to the NDA. Instead, only daily aggregates of key input data (e.g., daily word count) were submitted to the NDA.

**EEG Data Preprocessing Procedures**

Offline analyses were performed in MATLAB using customized scripts and the EEGLAB toolbox [[3]](https://www.zotero.org/google-docs/?2Kf7Bw). First, a band-pass filter was then applied from 1-100 Hz. The 60 Hz line noise was removed using the cleanLineNoise function, which removed line noise by estimating the noise profile through spectral decomposition and applying adaptive notch filtering [[4]](https://www.zotero.org/google-docs/?xNB0X0). Next, the clean_rawdata function corrected artifacts by automatically identifying and rejecting contaminated epochs based on amplitude and variance thresholds, defined as those: (a) containing more than 5s of flat signal, and/or (b) correlating less than .7 with surrounding channels [[5]](https://www.zotero.org/google-docs/?Jqacw5). Artifact subspace reconstruction (ASR) was then applied to correct significant noise bursts, also implemented within clean_rawdata [[6]](https://www.zotero.org/google-docs/?fjwAJp). ASR is a principal component analysis (PCA) based technique in which data within a 500 ms sliding window (window step=250 ms) were PCA-decomposed. Noisy components, defined as those with variance greater than 20SD above that of the clean portions of the data, were corrected. Last, independent component analysis (ICA) was implemented to retain brain-related components only, defined as having greater probability to be brain than artifacts according to an automatic independent component (IC) classifier (ICLabel) [[7]](https://www.zotero.org/google-docs/?Wdp3oE).

**References**

[1. Lind MN, Byrne ML, Wicks G, Smidt AM, Allen NB. The Effortless Assessment of Risk States (EARS) Tool: An Interpersonal Approach to Mobile Sensing. JMIR Ment Health. 2018;5:e10334.](https://www.zotero.org/google-docs/?iofjdo)

[2. Lind MN, Kahn LE, Crowley R, Reed W, Wicks G, Allen NB. Reintroducing the Effortless Assessment Research System (EARS). JMIR Ment Health. 2023;10:e38920.](https://www.zotero.org/google-docs/?iofjdo)

[3. Delorme A, Makeig S. EEGLAB: an open source toolbox for analysis of single-trial EEG dynamics including independent component analysis. J Neurosci Methods. 2004;134:9–21.](https://www.zotero.org/google-docs/?iofjdo)

[4. Bigdely-Shamlo N, Mullen T, Kothe C, Su K-M, Robbins KA. The PREP pipeline: standardized preprocessing for large-scale EEG analysis. Front Neuroinformatics. 2015;9.](https://www.zotero.org/google-docs/?iofjdo)

[5. Kothe CA, Makeig S. BCILAB: a platform for brain–computer interface development. J Neural Eng. 2013;10:056014.](https://www.zotero.org/google-docs/?iofjdo)

[6. T. Mullen, C. Kothe, Y. M. Chi, A. Ojeda, T. Kerth, S. Makeig, et al. Real-time modeling and 3D visualization of source dynamics and connectivity using wearable EEG. 2013 35th Annu. Int. Conf. IEEE Eng. Med. Biol. Soc. EMBC, 2013. p. 2184–2187.](https://www.zotero.org/google-docs/?iofjdo)

[7. Pion-Tonachini L, Kreutz-Delgado K, Makeig S. ICLabel: An automated electroencephalographic independent component classifier, dataset, and website. NeuroImage. 2019;198:181–197.](https://www.zotero.org/google-docs/?iofjdo)

**Supplementary Figure 1**. Group-level differences in effective connectivity (remMDD – HC) are plotted as t-scores. Each panel displays results for one frequency band (delta through gamma). Significant connections (p < .05, cluster-corrected) are shown in red. (A) Delta-band connectivity matrix (B) Theta-band connectivity matrix (C) Alpha-band connectivity matrix (D) Beta-band connectivity matrix (E) Gamma-band connectivity matrix


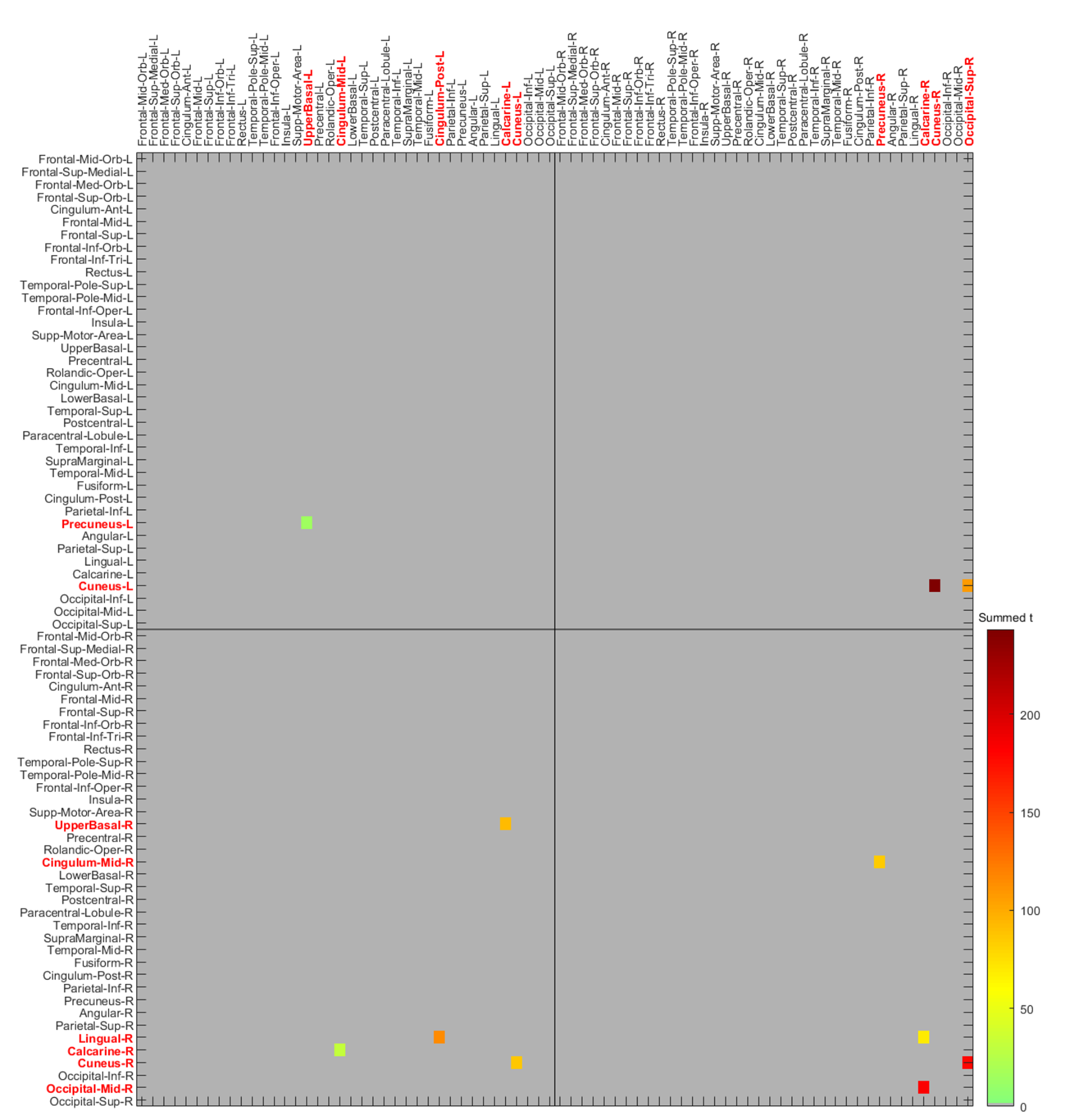


1. **Delta-band connectivity matrix**


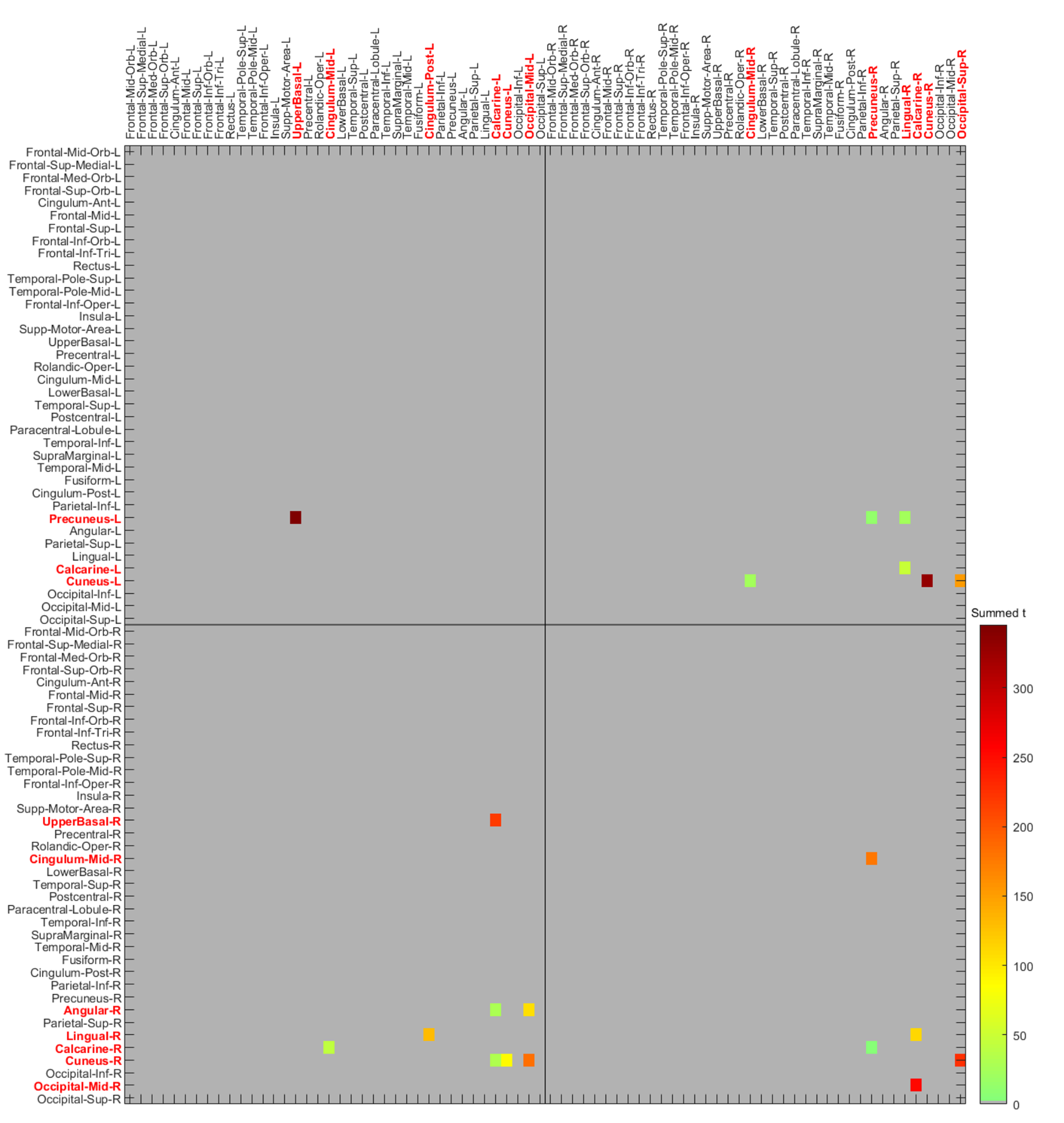


**(B) Theta-band connectivity matrix**


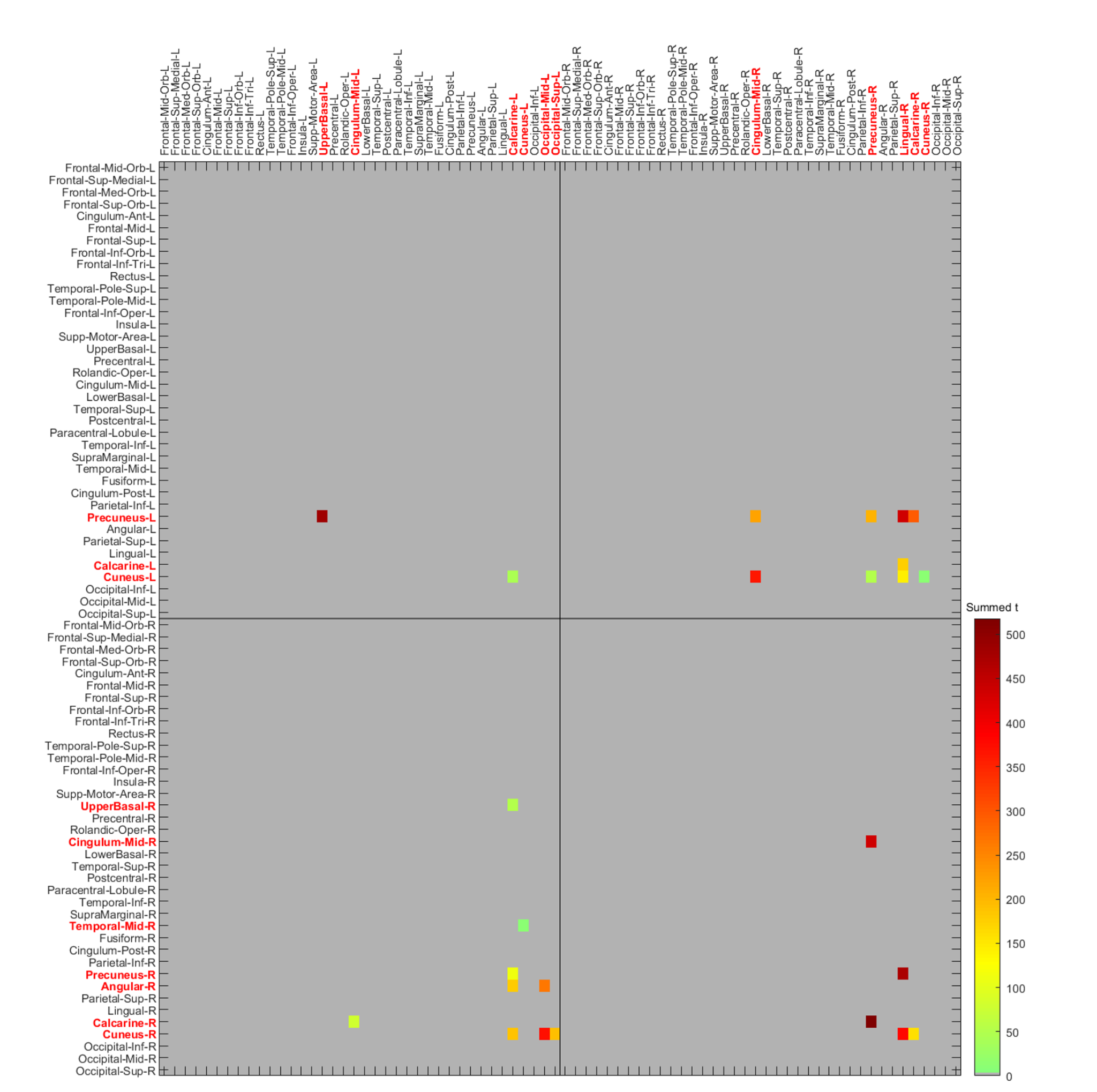


**(C) Alpha-band connectivity matrix**


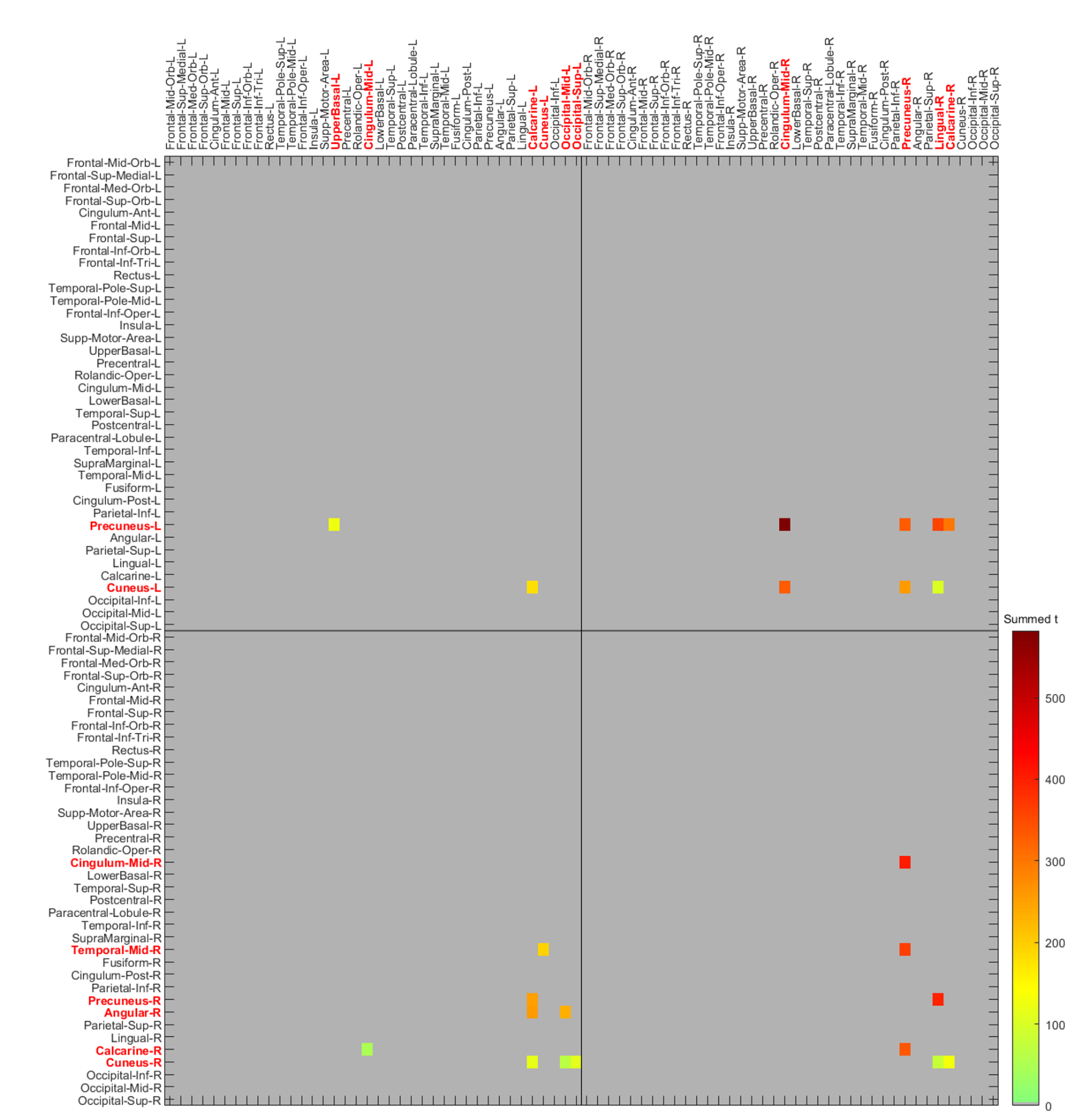


**(D) Beta-band connectivity matrix**


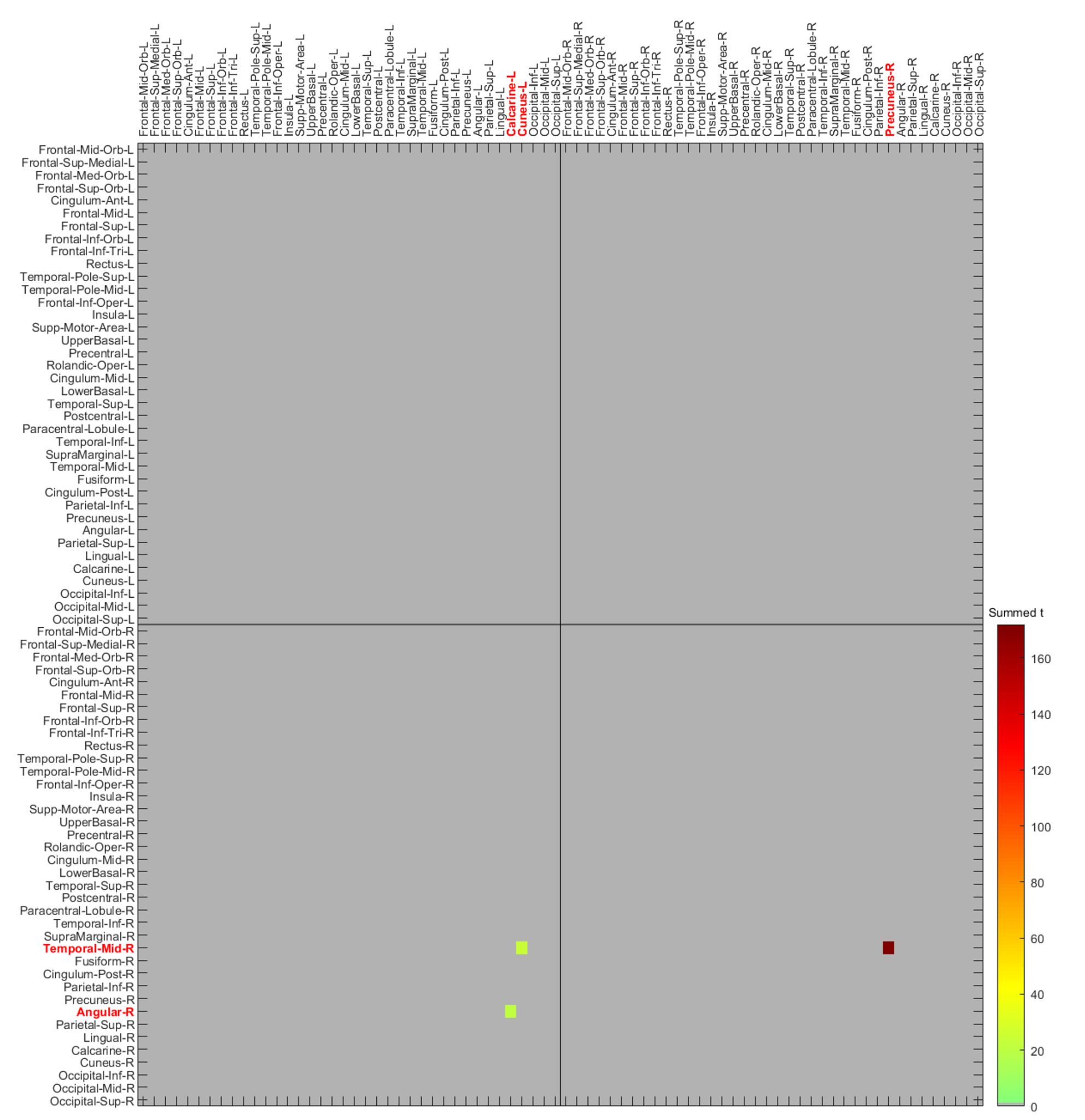


**(E) Gamma-band connectivity matrix**

**Supplementary Table 1.** Alpha band connectivity strength from mid cingulate cortex to precuneus predicting depression symptoms at the 6-month follow-up assessment

|  | Delta-band  (Precuneus→MCC) | | | Theta-band  (Precuneus→MCC) | | | Alpha-band  (MCC→Precuneus) | | | Beta-band  (MCC→Precuneus) | | |
| --- | --- | --- | --- | --- | --- | --- | --- | --- | --- | --- | --- | --- |
|  | **b** | **SE** | ***p*** | **b** | **SE** | ***p*** | **b** | **SE** | ***p*** | **b** | **SE** | ***p*** |
| (intercept) | 2.515 | 0.344 | <0.001 | 2.501 | 0.348 | <0.001 | 1.976 | 0.501 | <0.001 | 2.674 | 0.688 | <0.001 |
| Age | 0.018 | 0.021 | 0.389 | 0.017 | 0.021 | 0.399 | -0.004 | 0.024 | 0.873 | -0.003 | 0.0414 | 0.936 |
| Sex | -0.102 | 0.068 | 0.133 | -0.103 | 0.068 | 0.129 | -0.166 | 0.084 | 0.050 | -0.095 | 0.0637 | 0.135 |
| Site | -0.079 | 0.060 | 0.187 | -0.076 | 0.059 | 0.200 | -0.049 | 0.071 | 0.494 | -0.047 | 0.0561 | 0.404 |
| Baseline depressive symptoms | 0.014 | 0.007 | 0.044 | 0.014 | 0.007 | 0.042 | 0.024 | 0.007 | 0.001 | 0.014 | 0.006 | 0.025 |
| Baseline connectivity | -2.416 | 7.354 | 0.743 | 1.374 | 13.84 | 0.921 | -0.239 | 0.096 | 0.013 | 7.393 | 19.37 | 0.703 |

Note. MCC: mid cingulate cortex
